# Can work ability explain the social gradient in sickness absence: a study of a general population in Sweden

**DOI:** 10.1186/1471-2458-12-163

**Published:** 2012-03-07

**Authors:** Jesper Löve, Kristina Holmgren, Kjell Torén, Gunnel Hensing

**Affiliations:** 1 Department of Community Medicine and Public Health, The Sahlgrenska Academy, University of Gothenburg, Gothenburg, Sweden

## Abstract

**Background:**

Understanding the reasons for the social gradient in sickness absence might provide an opportunity to reduce the general rates of sickness absence. The complete explanation for this social gradient still remains unclear and there is a need for studies using randomized working population samples. The main aim of the present study was to investigate if self-reported work ability could explain the association between low socioeconomic position and belonging to a sample of new cases of sick-listed employees.

**Methods:**

The two study samples consisted of a randomized working population (n = 2,763) and a sample of new cases of sick-listed employees (n = 3,044), 19-64 years old. Both samples were drawn from the same randomized general population. Socioeconomic status was measured with occupational position and physical and mental work ability was measured with two items extracted from the work ability index.

**Results:**

There was an association between lower socioeconomic status and belonging to the sick-listed sample among both women and men. In men the crude Odds ratios increased for each downwards step in socioeconomic status, OR 1.32 (95% CI 0.98-1.78), OR 1.53 (1.05-2.24), OR 2.80 (2.11-3.72), and OR 2.98 (2.27-3.90). Among women this gradient was not as pronounced. Physical work ability constituted the strongest explanatory factor explaining the total association between socioeconomic status and being sick-listed in women. However, among men, the association between skilled non-manual, OR 2.07 (1.54-2.78), and non-skilled manual, OR 2.03 (1.53-2.71) positions in relation to being sick-listed remained. The explanatory effect of mental work ability was small. Surprisingly, even in the sick-listed sample most respondents had high mental and physical work ability.

**Conclusions:**

These results suggest that physical work ability may be an important key in explaining the social gradient in sickness absence, particularly in women. Hence, it is possible that the factors associated with the social gradient in sickness absence may differ, to some extent, between women and men.

## Background

Low socioeconomic status has consistently been associated with higher rates of sickness absence [1-9]. Manual labour workers tend to have two to three times more episodes of medically-certified sickness absence than managers and professionals [3,10]. Consequently, understanding the reasons for the social gradient in sickness absence might provide an opportunity to reduce the general rates of sickness absence. Attempts to explain the social gradient have mainly focused on the explanatory effect of physical and psychosocial working conditions, health-related behaviors, and health [1,3,7,9]. Recently, Christensen et al (2008) and Laaksonen et al (2010) in unison found that physical working conditions were the strongest explanatory factor for occupational class differences in sickness absence. The effect of health-related behavior seems to be less prominent [1,3], much like the effect of psychosocial work factors which seem to be small [1,2,7] or even reversed [7]. Beyond social and structural factors, studies examining self-rated general health as an explanatory factor show contradictory and inconclusive results [2,7,11].

It has been found that the association between socioeconomic status and sickness absence may vary by duration and pattern of sickness absence [7]. A recent study by Virtanen et al (2011) also suggests that this association may be diagnose-specific [12]. The association also tends to be stronger in men [1,2,7], and the significance of specific explanatory factors for this association may differ between women and men. For example, although the research on this matter is rather scarce Christensen et al (2008) found, in a random sample of Danish employees, that psychosocial work environment had some effect on the social gradient in women but not in men [1]. Although the social gradient in sickness absence is explained to some extent by the factors presented above, the complete explanation for this association still remains unclear. Moreover, since few studies have been conducted on randomized working population samples their external validity has been questioned, due to for example limitations in age span and job security [2]. Consequently, there is a need for studies on the social gradient of sickness absence and its explanatory factors, particularly using randomized working population samples.

The concept of Work ability is seen as the product of individual resources and work demands. As such, it is one of the main constituents of sickness absence [13]. One can assume that work ability may contribute to the social gradient in sickness absence since there is a social gradient even in work ability [14] and that work ability is a predictor of future sickness absence [15,16]. Thus, the mechanism would be that the decreased level of work ability among lower socioeconomic groups will eventually result in a higher proportion that become sick-listed than in higher socioeconomic groups where level of work ability is usually higher. Beyond the empirically based hypothesis that work ability may contribute to explain the social gradient in sickness absence, in Sweden there is also a policy based argument for conducting such an examination. The Swedish health insurance system affirms that decreased work ability is the main criterion for becoming sick-listed, although the decrease in work ability must be related to a specific disease or injury. Hence, the higher proportion of sick-listed in lower socioeconomic groups should mainly be an effect of decreased work ability.

To that end, the main aim of the present study was to investigate if self-reported work ability could explain the social gradient in sickness absence in women and men. This aim was examined through four research questions: 1) is there a distributional difference in socioeconomic status between an sample of newly sick-listed employees and a randomized working population sample, 2) if yes, does a distributional difference in socioeconomic status follow the same pattern for women and men, 3) can a potential social gradient in the sample of newly sick-listed be explained by differences in the level of work ability, 4) if yes, does the explanatory effect of work ability follow the same pattern for women and men.

## Methods

The study population consisted of the baseline data of an ongoing cohort study, i.e. The Health Assets Project. The main aim of this project was to study the influence of individual, organizational, and societal factors on health, sickness absence, and return to work. The Health Assets Project is based on the population of the Västra Götaland region of western Sweden, inhabiting approximately 1.6 million people and including both urban and rural areas. The project started in 2008 and comprises a randomized population sample and a sample of new cases of sick-listed employees, 19-64 years old. Consequently both samples are drawn from the same overall population. The randomized population sample was randomly selected by Statistics Sweden. The sick-listed sample was identified by The Swedish Social Insurance Agency as all employer-reported new cases of sick-leave (i.e. > 14 days), during a period from the 18^th ^of February 2008 to the 15^th ^of April 2008, in the Västra Götaland region. Consequently, these individuals became sick-listed in a new sick-leave spell and none of them had any earlier sick-leave spell the year under study. Baseline data was then collected from the 15^th ^of April 2008 to the 30^th ^of June 2008. Data was collected using registered data and a postal questionnaire including items on socio-demographic factors, physical and mental health, sick leave, working life, family conditions, life-events, leisure and lifestyle. At baseline, 2234 women (response rate 57%) and 1793 men (44%) in the randomized population sample, and 2196 women (58%) and 1114 men (47%) the sick-listed sample responded to the questionnaire. A drop-out analysis of the total study population showed a statistically significantly higher drop-out rate for women and men in the youngest age group (i.e. 19-30 years of age), in the lowest income level, ≤ 149 000 SEK, and among those born outside Sweden and the other Nordic countries. Separate analyses of each sample showed an overall similar pattern.

There was a delay between the time of inclusion of individuals in the project and the time when they answered the questionnaire. Since sickness absence is dynamic, some individuals in the randomized working population sample had entered sickness absence at the time of answering the questionnaire (5.6% of the study sample described below) while individuals in the sick-listed sample had returned to work (54.8% of the of the study sample described below). There is unfortunately no way of avoiding this time delay in a randomized population study. The Health Assets Project was approved by the Ethics Committee, University of Gothenburg, Sweden.

Only employed participants were included in the present study sample. Consequently, the study sample consisted of 5,807 employees of which 2,763 belonged to the randomized working population sample and 3,044 to the sample of newly sick-listed employees (Table 1).

**Table 1:** The procedure of study selection in the randomized working population sample and the newly sick-listed sample

Samples	**Participants in the Health Assets Project**, n (%)			**Participants (employed)**, n (%)		
	
	Women	Men	Total	Women	Men	Total
Randomized working population sample	2234 (55)	1793 (45)	4027	1525 (55)	1238 (45)	2763
Newly sick-listed sample	2521 (66)	1287 (34)	3838	2020 (66)	1022 (34)	3044
Total	4755 (61)	3080 (39)	7835	3547 (61)	2260 (39)	5807

### Demographic variables

Age was categorized into three groups: 19-30, 31-50, and 51-64 years. Income was categorized into three groups: 0-149 000, 150 000-299 000, and 300 000 Swedish Krona per year. Land of birth was categorized into two groups: Nordic countries and other. Educational level had six levels that were categorized into three groups: Primary education, Secondary education, and University or College. Respondents on sick-leave also reported the medical cause for them currently being on sick-leave. It was here possible to tick several alternatives of the twelve response alternatives and the one open text alternative.

### Explanatory variables

Socioeconomic status was measured with occupational position based on the definitions of Statistics Sweden [17]. Initially, six levels of socioeconomic status were applied: higher non-manual employees, intermediate non-manual employees, lower non-manual employees, skilled worker, unskilled worker, entrepreneurs. However, because only three individuals were classified as being entrepreneurs these individuals were included in the higher non-manual employees' category.

Since the demands at work differ between manual and non-manual workers it may also be important to analyze the effect of mental and physical work ability independently.

Hence, work ability was measured with two items extracted from the work ability index: *How do you rate your current work ability with respect to the mental demands of your work, and How do you rate your current work ability with respect to the physical demands of your work? *The Work Ability Index response scale was employed with five ordinal alternatives: *very good, rather good, moderate, rather poor, very poor*. According to the psychometric evaluation of the Work Ability Index the two items used in the present study show one of the highest correlations with the total index [18]. The items were dichotomized as high (i.e. very good, rather good) and low (i.e. moderate, rather poor, very poor). The Work ability index have previously shown high construct validity [18], satisfying test-retest reliability [19], and prediction of work disability, retirement and mortality [20].

### Outcome variables

The main outcome was defined as the odds ratio of belonging to the sample of newly sick listed with the randomized working population sample as reference.

### Statistical analyses

Analyses of proportions and their differences were calculated using Confidence interval analyses outlined in Altman et al [21]. All other calculations were run using SAS version 9.2 (SAS Institute, Cary, NC).

Distributional differences in socio-demographic characteristics between the randomized working population sample and the sample of newly sick-listed were calculated with a number of chi-square tests (i.e. age, income, land of birth, educational level, occupational position, employer, physical work ability, mental work ability, hours worked/week, current sick leave). Chi-square tests were also used when examining the relation between socioeconomic status and the proportion belonging to the sick-listed sample or the randomized working population sample, for women and men separately. In examining the explanatory effect of work ability a series of logistic regression analyses were calculated in the total population and in women and men respectively. The LOGISTIC procedure in SAS was utilized. Socioeconomic status was inserted as a CLASS variable resulting in a point estimate for all socioeconomic status levels respectively, with higher non-manual as reference. First, the bivariate association between different levels of socioeconomic status and being sick-listed was examined by calculating the Odds ratios (OR) and 95% Confidence intervals for belonging to the sick-listed sample. Thereafter, mental and physical work ability was added in two separate models. The explanatory effect of work ability was then evaluated through comparing the size of the point estimates (OR) and width of the Confidence interval with the point estimates of the bivariate analyses. As mentioned above, the delay between the time of inclusion and the time when the respondents answered the questionnaire allowed individuals in the sick-listed sample to go back to work, and individuals in the randomized working population sample to enter a period of sickness absence. To address this issue, additional analyses were conducted on a more homogenous sample. In these analyses all individuals belonging to the randomized working population sample that had entered a period of sickness absence, and all individuals belonging to the sick-listed sample that had returned to work, were deleted.

## Results

The randomized working population sample had a lower proportion of manual workers and a larger proportion of non-manual workers, in comparison to the employed sick-listed sample. This distributional difference between the samples was more pronounced in men regarding age and education, and in women regarding being privately or publically employed. In both the randomized working population sample and the sick-listed sample a majority reported high mental and physical work ability, in both women and men. Yet, the proportion with high physical and mental work ability was larger in the randomized working population sample (Table 2). In the additional analyses, on the more homogenous study samples, the proportion having high mental and physical work ability in the sick-listed sample decreased but was still larger (*p *< 0.0001) than the proportion reporting low work ability in this sample. The great majority reported that they were sick-listed due to Common mental disorders and/or musculoskeletal disorders. Only very few reported infectious disease (figures not shown).

**Table 2:** Characteristics of the participants in the randomized working population sample and the sick-listed sample (n = 5807), for women and men respectively

	Women n = 3547			Men n = 2260		
	
	Sick-listed sample n = 2022	Pearson's Chi square^1^	Randomized working population sample n = 1525	Sick-listed sample n = 1022	Pearson's Chi square^1^	Randomized working population sample n = 1238
	
	% (n)	**Chisq**. Value	% (n)	% (n)	**Chisq**. Value	% (n)
**Years old**						
19-30	10 (208)*		15 (224)	12 (125)*		17 (215)
31-50	47 (953)	23.91	49 (751)	40 (412)*	47.75	49 (610)
51-64	43 (861)*		36 (550)	48 (485)*		34 (413)
**Income**						
0-149 000	10 (209)*		15 (230)	6 (67)*		10 (124)
150 000-299 000	73 (1478)*	43.71	63 (957)	57 (580)*	48.05	42 (523)
300 000-	17 (335)*		22 (338)	37 (375)*		48 (591)
**Land of birth**						
Nordic countries	92 (1857)	3.23	93 (1425)	88 (896)*	7.10	91 (1128)
Other	8 (165)		7 (100)	12 (126)*		9 (110)
**Educational level**						
Primary education	20 (393)*		16 (245)	31 (309)*		17 (210)
Secondary education	40 (801)	8.85	40 (598)	49 (489)	83.42	48 (594)
University or college	40 (805)*		44 (668)	20 (207)*		35 (427)
**Socioeconomic position**						
Higher non-manual	11 (227)*		16 (234)	11 (106)*		21 (257)
Intermediate non-manual	27 (550)		30 (447)	16 (158)*		24 (290)
Lower non-manual	15 (291)*	86.89	18 (265)	7 (67)*	101.23	9 (290)
Skilled manual	21 (425)*		17 (252)	29 (288)*		21 (249)
Non-skilled manual	26 (511)*		20 (303)	38 (383)*		26 (312)
**Mental work ability**						
High	75 (1479)*	86.89	88 (1313)	78 (753)*	66.13	90 (1098)
**Physical work ability**						
High	65 (1272)*	214.13	87 (1293)	69 (675)*	139.85	90 (1085)
**Employer**						
Private/self-employed	30 (567)*	36.52	40 (576)	71 (672)	3.20	73 (856)
Public	70 (1332)*		60 (855)	29 (269)		27 (317)
**Current sick-leave**						
Yes	46 (924)*	630.93	7 (107)	44 (447)*	519.64	4 (48)

*Statistically significant difference (*p *< 0.05), between the sick-listed sample and the randomized working population sample

^1 ^Chi square value for the distributional comparison between the sick-listed sample and the randomized working population sample

Women were more likely than men to be sick-listed in all socioeconomic groups respectively, particularly in lower socioeconomic-groups. The Odds ratio of women in relation to men were, for higher non-manual, OR 1.37 (1.12-1.69), intermediate non-manual, OR 1.46 (1.16-1.84), lower non-manual OR 1.74 (1.23-2.46), skilled manual, OR 2.26 (1.79-2.84), and un-skilled manual, OR 2.35 (1.76-3.15).

The lower socioeconomic groups had a higher proportion belonging to the sick-listed sample, among both women and men. Nevertheless, the relation between socioeconomic status and belonging to the sick-listed sample showed somewhat different patterns for women and men. Among women and higher non-manuals there were equal proportions (*p *< 0.05) belonging to the sick-listed sample as to the randomized working population sample. Among men on the other hand, 71% of the higher non-manuals belonged to the randomized working population sample. This pattern was evident also among intermediate non-manuals and lower non-manuals. The opposite pattern was observable in the lower socioeconomic groups where the proportion of sick-listed was 63% for skilled manuals among women whereas among men having an equal proportion in each sample (Table 3).

**Table 3:** Distribution of belonging to the sick-listed sample or the randomized general working population for different socioeconomic groups.

	Women n = 3505		Men n = 2216	
	
Socioeconomic status	Newly sick-listed sample n = 2004	Randomized working population sample n = 1501	Newly sick-listed sample n = 1002	Randomized working population sample n = 1214
Higher non-manual	49 (227)	51 (234)	29 (106)*	71 (257)
Intermediate non-manual	55 (550)*	45 (447)	35 (158)*	65 (290)
Lower non-manual	52 (291)	48 (265)	39 (67)*	61 (106)
Skilled manual	63 (425)*	37 (252)	54 (288)	46 (249)
Non-skilled manual	63 (511)*	37 (303)	55 (383)*	45 (312)

Presented for women and men separately, % (n)

*Statistically significant difference (*p *< 0.05), between the sick-listed sample and the randomized working population sample

There was an association between lower socioeconomic status and belonging to the sick-listed sample among both women and men. All Or's calculated with higher non-manual as reference. In men the crude Odds ratios (i.e. Model 1) increased for each downwards step in socioeconomic status, OR 1.32 (0.98-1.78), OR 1.53 (1.05-2.24), OR 2.80 (2.11-3.72), and OR 2.98 (2.27-3.90). Among women this gradient was not as pronounced. That is, although there was an association for intermediate non-manuals OR 1.27 (1.02-1.58) there was no association observed for lower non-manuals, OR 1.13 (0.88-1.45), and skilled and un-skilled manual positions did not differ in size of the point estimate with, OR 1.74 (1.37-2.21), and OR 1.74 (1.40-2.19) respectively. When entering mental work ability (i.e. Model 2) the changes in point estimates and confidence intervals were small compared to the crude analyses (i.e. Model 1). However, when entering physical work ability (i.e. model 3) all associations between socioeconomic status and belonging to the sick-listed sample became statistically insignificant (95% CI including 1), among women. Among men, the association between lower, intermediate, and higher non-manual positions became statistically insignificant (i.e. model 3). Yet, the association between skilled non-manual, OR 2.07 (1.54-2.78), and non-skilled manual, OR 2.03 (1.53-2.71) positions in relation to being sick-listed remained (Table 4). In the additional analyses on the more homogenous study sample the social gradient was somewhat more pronounced among women than they were in the main analyses. As in the main analyses the gradient were even more pronounced in men. Mental work ability had also a somewhat higher explanatory effect in the additional analyses, although the associations between non-manual positions and being sick-listed remained in both women and men. As in the main analyses, when entering physical work ability all associations between socioeconomic status and being sick-listed disappeared, among women. Yet, in line with the main analyses the association between non-manual positions and being sick-listed remained, among men.

**Table 4:** Logistic multivariate regressions between lower levels of socioeconomic status and belonging to the sick-listed sample, with higher non-manual as reference.

	Women (n = 3547)			Men (n = 2260)		
	
	Model 1 OR (95% CI)	Model 2 OR (95% CI)	Model 3 OR (95% CI)	Model 1 OR (95% CI)	Model 2 OR (95% CI)	Model 3 OR (95% CI)
Higher non-manual	1.00	1.00	1.00	1.00	1.00	1.00
Intermediate non-manual	1.27 (1.02-1.58)	1.29 (1.03-1.62)	1.13 (0.89-1.42)	1.32 (0.98-1.78)	1.30 (0.96-1.76)	1.22 (0.90-1.66)
Lower non-manual	1.13 (0.88-1.45)	1.21 (0.94-1.56)	1.05 (0.81-1.35)	1.53 (1.05-2.24)	1.52 (1.03-2.25)	1.31 (0.89-1.95)
Skilled manual	1.74 (1.37-2.21)	1.84 (1.44-2.35)	1.19 (0.93-1.53)	2.80 (2.11-3.72)	2.82 (2.11-3.76)	2.07 (1.54-2.78)
Non-skilled manual	1.74 (1.40-2.19)	1.82 (1.43-2.30)	1.27 (0.99-1.64)	2.98 (2.27-3.90)	2.75 (2.08-3.64)	2.03 (1.53-2.71)
(Low) mental work ability		1.46 (1.35-1.58)			1.53 (1.37-1.70)	
(Low) physical work ability			1.75 (1.62-1.89)			1.70 (1.54-1.88)

Crude Odds ratios (OR) with 95% Confidence intervals (CI) are presented in *Model 1*. The explanatory effect of work ability is examined in *Model 2 *(mental work ability) and *Model 3 *(physical work ability)

## Discussion

There was an evident association between socioeconomic status and being sick-listed in the present study. Although this finding is in line with several previous studies [1-3,7,9,11] this is the first study observing a social gradient in sickness absence using new cases of sick-listed employees (> 14 days) in a Swedish randomized working population sample. Since there has been a need for more studies conducted on randomized working population samples [2] the present findings provide an important contribution to previous findings in Norway [2] and in Denmark [1]. The association between socioeconomic status and being sick-listed was observed in both women and men. Yet, it seemed to be stronger in men. Physical work ability constituted the strongest explanatory factor explaining the total association between socioeconomic status and being sick-listed in women. However, among men, a substantial part of this association remained unexplained. The explanatory effect of mental work ability was small. Surprisingly, even in the sick-listed sample most respondents had high mental and physical work ability.

The association between socioeconomic status and sickness absence was present in both women and men. However, albeit comparing Odds ratios from stratified analyses should be done with great caution, when taking these comparisons into account in combination with the proportional distribution the association was somewhat stronger in men. In addition, the specific gradients between different socioeconomic groups were also more pronounced in men. This pattern was particularly evident among men in the logistic regression analyses where the size of the point estimates increased for each downward level of socioeconomic status. Yet, the social gradient was somewhat more pronounced for women in the additional analyses on the more homogenous study sample, why the differences in the main analyses should be interpreted with some caution. Still the difference between women and men persisted and even in the additional analyses the gradient was more pronounced among men. That the social gradient was more prominent in men is in line with a range of studies on various health outcomes but also with a few previous studies on sickness absence [2,3]. It has been suggested that measures of socioeconomic status may have less precision in women since they fail to capture gender as a significant element of these structures [22-24]. This hypothesis also correspond with Koskinen and Martelin (1994) suggesting that the confounding influence of other socio-demographic variables, may either mask inequities in women or accentuate them in men [25]. Hence, it is possible that the use of occupational class as a measure of socioeconomic status may have accentuated the observed difference between women and men in the present study. Yet, these differences may also mirror the vertical gender segregation [26] resulting in highly educated women more often working in lower occupational positions than men with the same level of education. Moreover, although being categorized in the same occupational class women and men most often work in different occupational fields [27] which sequentially end up in divergent patterns of exposures. In sum, it is possible that the measure of occupational class may not represent the same social structure in both women and men. Thus, a relevant question would be whether the usage of another measure of socioeconomic status would result in divergent findings. However, that substantial co-variations between occupational class, educational level, and income, were found in the present data in both women and men (figures not shown) give that a potential bias compared to using educational level or income for measuring socioeconomic status should be small. Still, future studies should analyze the effect of different measures of socioeconomic status on the social gradient of sickness absence for both women and men.

There was a strong explanatory effect of physical work ability on the association between socioeconomic status and sickness absence in the present study. While previous studies have mainly focused on physical and psychosocial work conditions this is the first study pointing towards the importance of work ability in explaining the social gradient in sickness absence. These results then complement previous research by Laaksonen and colleagues (2010) and Hansen and Ingebrigtsen (2008) showing that physical work conditions provide the strongest explanation for the social gradient in sickness absence [2,3]. Consequently, the present findings might support a proposed mechanism comprising that a decreased level of work ability among lower socioeconomic groups will eventually result in a higher proportion that become sick-listed than in higher socioeconomic groups where level of physical work ability is usually higher. Since self-reported work ability is the product of both individual resources and work demands it is not possible to conclude whether the social gradient in physical work ability reflects higher physical work demands faced by the manual workers or their potentially lower individual resources. Still, since previous studies have found a social gradient in both physical health [28] and physical work demands [3] it is plausible to think that both these variables work in the same direction. Yet, we do not know whether they represent two independent effects or if an interaction effect may arise amid them.

The explanatory effect of mental work ability was almost negligible, in the main analyses. In the additional analyses on the more homogenous sample mental work ability has a somewhat stronger explanatory effect although most associations remained unexplained in both women and men. Yet, the result that mental work ability has lower explanatory effect may match previous findings showing that the explanatory effect of psychosocial work conditions on the social gradient of sickness absence is generally small [2,3]. That physical work ability was the most important explanatory factor also parallels the fact that there was a social gradient in physical but not in mental work ability in the present study (figures not shown). A social gradient in work ability has previously been observed using the work ability index [14]. Yet, we have found no such studies providing separate analyses for physical and mental work ability respectively. One may hypothesize that while it is probable that lower socioeconomic groups are more exposed to detrimental physical work conditions as a group there is little evidence that individual physical resources should differ in their favor. Consequently, we see a social gradient in physical work ability. On the other hand, it is much harder to hypothesize on the mechanism behind the non-existent social gradient in mental work ability. The main reason is that it is more difficult to interpret what the respondents put into their evaluation of mental work ability. For example, although the requirements of high competence and knowledge among employees in higher socioeconomic positions could also end up in high mental work demands, these demands are often accompanied with higher work control in turn balancing the effect of high demands [29].

In women, the association between socioeconomic status and sickness absence was fully explained by differences in physical work ability. However, among men, a substantial part of the difference between non-manual and manual positions in relation to being sick-listed remained unexplained. These results were true also in the additional analyses on the more homogenous study sample. That the social gradient in sickness absence is explained in women but not in men is in line with previous findings by Laaksonen et al (2010) and Hansen and Ingebrigtsen (2008). These issues is further discussed above and may mirror that due to horizontal and vertical gender segregation measures of socioeconomic status may not reflect the same living conditions in women as in men. For example, the association between socioeconomic status and being sick-listed was less pronounced in women. That the association between socioeconomic status and sickness absence was explained in women but not in men may also suggest that we failed to cover factors important for this gradient in men. Another explanation may be that the items on work ability are perceived and, answered in heterogeneous ways by women and men. It is important to note that although reduced work ability is one major criterion for attaining a sick-leave certificate in Sweden; neither mental nor physical work ability explained the total association between socioeconomic status and sickness absence in men. So, are men in Sweden sick-listed for other reasons than reduced work ability? If this is not the case, one could question whether the two items from the Work Ability Index may measure different things in a healthy population and in a sick-listed population and that the items works better in women. We have not found any previous studies suggesting that this would be the case. Still, it is plausible to think that since only newly sick-listed cases were included in the present study they may answer the work ability items with their normal work ability in mind, particularly if the reason for them being on sick-leave is a health problem with short duration. On the other hand, the issue of whether the items are as valid in men as in women persists. In preliminary analyses we have also seen that work ability had different explanatory effect, for being sick-listed, in different socioeconomic groups. This will be furthermore examined in an upcoming study.

That the vast majority even in the sick-listed sample had a high level of work ability imply that the relation between being sick-listed and work ability should be further analyzed in future studies. Particularly, since level of work ability is the main constituent when evaluating individual's right to sickness benefit in Sweden.

One of the strengths of this study is that the measure of sickness absence was not based on self-reports but on new cases of sick-listed employees (< 14 days), reported by the employer. On the other hand this strength could also have worked as a limitation since the delay between inclusion and the respondents filling out the questionnaire allow some of them to go back to work. This give that the ones in the sick-listed sample that had already gone back to work had probably had also retained their work ability. There is unfortunately no way of getting around this problem when using new sick-leave cases but in order to address this limitation, additional analyses were conducted where all individuals not currently being sick-listed were deleted from the study sample (presented in the result section and in the discussion above). Although some differences appeared the results of the additional analyses were in line with the main analyses. That the study was conducted on a randomized working population sample also provide an important contribution to the literature about the social gradient in sickness absence. However, since the two measures on work ability were self-reports interpretations of their explanatory effects have to be made with some caution. It is also possible that their single-item design may have contributed to a lower reliability. Still, these two items are retained from the well-used Work ability index and has previously shown high reliability and validity [18]. Unfortunately, we had no information on the diagnosed cause for being sick-listed. Still, according to a recent publication [12] this information could have provided important information even in the present study. The overall response rate was rather low (i.e. 52%) and the analysis on non-respondents suggest that in line with most similar studies the proportions of men, younger individuals, and individuals born outside the Nordic countries are lower than in the total population. Finally, although the present study provide important and complementing knowledge we acknowledge that more complicated methodology may be needed to provide more accurate answers in the question of the social gradient in sickness absence (e.g. see [30]).

## Conclusions

There was an evident association between socioeconomic status and sickness absence in this study. This social gradient seemed stronger, and the specific gradient between different socioeconomic groups was more pronounced, in men than in women. Physical work ability constituted the strongest explanatory factor explaining the total association between socioeconomic status and being sick-listed in women. Nonetheless, in men, the gradient between manual and non-manual workers remained unexplained. The explanatory effect of mental work ability was small in both women and men. Surprisingly, even in the sick-listed sample most respondents had high mental and physical work ability.

By using a Swedish randomized working population sample and employer-reported new cases of sick-listed employees the present study further strengthen the evidence of a social gradient in sickness absence. Yet, the findings also provide additional indications that this social gradient show different patterns in women and in men. That self-reported physical work ability was a strong explanatory factor implies that the mechanism behind this social gradient is partly due to a social gradient in work ability, particularly among women. However, since physical work ability did not explain the social gradient between manual and non-manual employees in men further studies should identify other factors of importance for this association among men.

## Abbreviations

OR: Odds ratios; CI: Confidence intervals.

## Competing interests

The authors declare that they have no competing interests.

## Authors' contributions

All authors participated in the design of the study. GH came up with the idea and the design of the overall project. GH is also responsible for the content of the questionnaire. KH was very active in the construction of the project and the data collection JL performed the statistical analyses and drafted the manuscript. KH, GH, and KT contributed continuously to the manuscript throughout the writing process. All authors read and approved the final manuscript.

## Pre-publication history

The pre-publication history for this paper can be accessed here:

http://www.biomedcentral.com/1471-2458/12/163/prepub
